# Asymptomatic and Presymptomatic SARS-CoV-2 Infections in Residents of a Long-Term Care Skilled Nursing Facility — King County, Washington, March 2020

**DOI:** 10.15585/mmwr.mm6913e1

**Published:** 2020-04-03

**Authors:** Anne Kimball, Kelly M. Hatfield, Melissa Arons, Allison James, Joanne Taylor, Kevin Spicer, Ana C. Bardossy, Lisa P. Oakley, Sukarma Tanwar, Zeshan Chisty, Jeneita M. Bell, Mark Methner, Josh Harney, Jesica R. Jacobs, Christina M. Carlson, Heather P. McLaughlin, Nimalie Stone, Shauna Clark, Claire Brostrom-Smith, Libby C. Page, Meagan Kay, James Lewis, Denny Russell, Brian Hiatt, Jessica Gant, Jeffrey S. Duchin, Thomas A. Clark, Margaret A. Honein, Sujan C. Reddy, John A. Jernigan, Public Health – Seattle & King County, CDC COVID-19 Investigation Team, Public Health – Seattle & King County, Atar Baer, Leslie M. Barnard, Eileen Benoliel, Meaghan S. Fagalde, Jessica Ferro, Hal Garcia Smith, Elysia Gonzales, Noel Hatley, Grace Hatt, Michaela Hope, Melinda Huntington-Frazier, Vance Kawakami, Jennifer L. Lenahan, Margaret D. Lukoff, Emily B. Maier, Shelly McKeirnan, Patricia Montgomery, Jennifer L. Morgan, Laura A. Mummert, Sargis Pogosjans, Francis X. Riedo, Leilani Schwarcz, Daniel Smith, Steve Stearns, Kaitlyn J. Sykes, Holly Whitney, CDC COVID-19 Investigation Team, Hammad Ali, Michelle Banks, Arun Balajee, Eric J. Chow, Barbara Cooper, Dustin W. Currie, Jonathan Dyal, Jessica Healy, Michael Hughes, Temet M. McMichael, Leisha Nolen, Christine Olson, Agam K. Rao, Kristine Schmit, Noah G. Schwartz, Farrell Tobolowsky, Rachael Zacks, Suzanne Zane

**Affiliations:** ^1^CDC COVID-19 Investigation Team; ^2^Epidemic Intelligence Service, CDC; ^3^Laboratory Leadership Service, CDC; ^4^Public Health – Seattle & King County; ^5^Washington State Public Health Laboratory.; Public Health – Seattle & King County; Public Health – Seattle & King County; Public Health – Seattle & King County; Public Health – Seattle & King County; Public Health – Seattle & King County; Public Health – Seattle & King County; Public Health – Seattle & King County; Public Health – Seattle & King County; Public Health – Seattle & King County; Public Health – Seattle & King County; Public Health – Seattle & King County; Public Health – Seattle & King County; Public Health – Seattle & King County; Public Health – Seattle & King County; Public Health – Seattle & King County; Public Health – Seattle & King County; Public Health – Seattle & King County; Public Health – Seattle & King County; Public Health – Seattle & King County; Public Health – Seattle & King County; Public Health – Seattle & King County; Public Health – Seattle & King County; Public Health – Seattle & King County; Public Health – Seattle & King County; Public Health – Seattle & King County; Public Health – Seattle & King County.; CDC; CDC; CDC; CDC; CDC; CDC; CDC; CDC; CDC; CDC; CDC; CDC; CDC; CDC; CDC; CDC; CDC; CDC.

Older adults are susceptible to severe coronavirus disease 2019 (COVID-19) outcomes as a consequence of their age and, in some cases, underlying health conditions (*1*). A COVID-19 outbreak in a long-term care skilled nursing facility (SNF) in King County, Washington that was first identified on February 28, 2020, highlighted the potential for rapid spread among residents of these types of facilities (*2*). On March 1, a health care provider at a second long-term care skilled nursing facility (facility A) in King County, Washington, had a positive test result for SARS-CoV-2, the novel coronavirus that causes COVID-19, after working while symptomatic on February 26 and 28. By March 6, seven residents of this second facility were symptomatic and had positive test results for SARS-CoV-2. On March 13, CDC performed symptom assessments and SARS-CoV-2 testing for 76 (93%) of the 82 facility A residents to evaluate the utility of symptom screening for identification of COVID-19 in SNF residents. Residents were categorized as asymptomatic or symptomatic at the time of testing, based on the absence or presence of fever, cough, shortness of breath, or other symptoms on the day of testing or during the preceding 14 days. Among 23 (30%) residents with positive test results, 10 (43%) had symptoms on the date of testing, and 13 (57%) were asymptomatic. Seven days after testing, 10 of these 13 previously asymptomatic residents had developed symptoms and were recategorized as presymptomatic at the time of testing. The reverse transcription–polymerase chain reaction (RT-PCR) testing cycle threshold (Ct) values indicated large quantities of viral RNA in asymptomatic, presymptomatic, and symptomatic residents, suggesting the potential for transmission regardless of symptoms. Symptom-based screening in SNFs could fail to identify approximately half of residents with COVID-19. Long-term care facilities should take proactive steps to prevent introduction of SARS-CoV-2 (*3*). Once a confirmed case is identified in an SNF, all residents should be placed on isolation precautions if possible (*3*), with considerations for extended use or reuse of personal protective equipment (PPE) as needed (*4*).

Immediately upon identification of the index case in facility A on March 1, nursing and administrative leadership instituted visitor restrictions, twice-daily assessments of COVID-19 signs and symptoms among residents, and fever screening of all health care personnel at the start of each shift. On March 6, Public Health – Seattle and King County, in collaboration with CDC, recommended infection prevention and control measures, including isolation of all symptomatic residents and use of gowns, gloves, eye protection, facemasks, and hand hygiene for health care personnel entering symptomatic residents’ rooms. A data collection tool was developed to ascertain symptom status and underlying medical conditions for all residents.

On March 13, the symptom assessment tool was completed by facility A’s nursing staff members by reviewing screening records of residents for the preceding 14 days and by clinician interview of residents at the time of specimen collection. For residents with significant cognitive impairment, symptoms were obtained solely from screening records. A follow-up symptom assessment was completed 7 days later by nursing staff members. Nasopharyngeal swabs were obtained from all 76 residents who agreed to testing and were present in the facility at the time; oropharyngeal swabs were also collected from most residents, depending upon their cooperation. The Washington State Public Health Laboratory performed one-step real-time RT-PCR assay on all specimens using the SARS-CoV-2 CDC assay protocol, which determines the presence of the virus through identification of two genetic markers, the N1 and N2 nucleocapsid protein gene regions (*5*). The Ct, the cycle number during RT-PCR testing when detection of viral amplicons occurs, is inversely correlated with the amount of RNA present; a Ct value <40 cycles denotes a positive result for SARS-CoV-2, with a lower value indicating a larger amount of viral RNA.

Residents were assessed for stable chronic symptoms (e.g., chronic, unchanged cough) as well as typical and atypical signs and symptoms of COVID-19. Typical COVID-19 signs and symptoms include fever, cough, and shortness of breath (*3*); potential atypical symptoms assessed included sore throat, chills, increased confusion, rhinorrhea or nasal congestion, myalgia, dizziness, malaise, headache, nausea, and diarrhea. Residents were categorized as asymptomatic (no symptoms or only stable chronic symptoms) or symptomatic (at least one new or worsened typical or atypical symptom of COVID-19) on the day of testing or during the preceding 14 days. Residents with positive test results and were asymptomatic at time of testing were reevaluated 1 week later to ascertain whether any symptoms had developed in the interim. Those who developed new symptoms were recategorized as presymptomatic. Ct values were compared for the recategorized symptom groups using one-way analysis of variance (ANOVA) for all residents with positive test results for SARS-CoV-2. Analyses were conducted using SAS statistical software (version 9.4; SAS Institute).

On March 13, among the 82 residents in facility A; 76 (92.7%) underwent symptom assessment and testing; three (3.7%) refused testing, two (2.4%) who had COVID-19 symptoms were transferred to a hospital before testing, and one (1.2%) was unavailable. Among the 76 tested residents, 23 (30.3%) had positive test results.

Demographic characteristics were similar among the 53 (69.7%) residents with negative test results and the 23 (30.3%) with positive test results (Table 1). Among the 23 residents with positive test results, 10 (43.5%) were symptomatic, and 13 (56.5%) were asymptomatic. Eight symptomatic residents had typical COVID-19 symptoms, and two had only atypical symptoms; the most common atypical symptoms reported were malaise (four residents) and nausea (three). Thirteen (24.5%) residents who had negative test results also reported typical and atypical COVID-19 symptoms during the 14 days preceding testing.

**TABLE 1:** Demographics and reported symptoms for residents of a long-term care skilled nursing facility at time of testing* (N = 76), by SARS-CoV-2 test results — facility A, King County, Washington, March 2020

Characteristic	Initial SARS-CoV-2 test results	
	Negative, no. (%)	Positive, no. (%)
**Overall**	53 (100)	23 (100)
Women	32 (60.4)	16 (69.6)
Age, mean (SD)	75.1 (10.9)	80.7 (8.4)
Current smoker^†^	7 (13.2)	1 (4.4)
Long-term admission type to facility A	35 (66.0)	15 (65.2)
Length of stay in facility A before test date, days, median (IQR)	94 (40–455)	70 (21–504)
**Symptoms in last 14 days**		
**Symptomatic**	13 (24.5)	10 (43.5)
At least one typical COVID-19 symptom^§^	9 (17.0)	8 (34.8)
Only atypical COVID-19 symptoms^¶^	4 (7.5)	2 (8.7)
**Asymptomatic**	40 (75.5)	13 (56.5)
No symptoms	32 (60.4)	8 (34.8)
Only stable, chronic symptoms	8 (15.1)	5 (21.7)
**Specific signs and symptoms reported as new or worse in last 14 days**		
**Typical symptoms**		
Fever	3 (5.7)	1 (4.3)
Cough	6 (11.3)	7 (30.4)
Shortness of breath	0 (0)	1 (4.4)
**Atypical symptoms**		
Malaise	1 (1.9)	4 (17.4)
Nausea	0 (0)	3 (13.0)
Sore throat	2 (3.8)	2 (8.7)
Confusion	2 (3.8)	1 (4.4)
Dizziness	1 (1.9)	1 (4.4)
Diarrhea	3 (5.7)	1 (4.4)
Rhinorrhea/Congestion	1 (1.9)	0 (0)
Myalgia	0 (0)	0 (0)
Headache	0 (0)	0 (0)
Chills	0 (0)	0 (0)
**Any preexisting medical condition listed**	53 (100)	22 (95.7)
Specific conditions**		
Chronic lung disease	16 (30.2)	10 (43.5)
Diabetes	20 (37.7)	9 (39.1)
Cardiovascular disease	36 (67.9)	20 (87.0)
Cerebrovascular accident	19 (35.9)	8 (34.8)
Renal disease	18 (34.0)	9 (39.1)
Received hemodialysis	2 (3.8)	2 (8.7)
Cognitive Impairment	28 (52.8)	13 (56.5)
Obesity	11 (20.8)	6 (26.1)

**Abbreviations:** COVID-19 = coronavirus disease 2019; IQR = interquartile range, SD = standard deviation.

* Testing performed on March 13, 2020.

^†^ Unknown for one resident with negative test results.

^§^ Typical symptoms include fever, cough, and shortness of breath.

^¶^ Atypical symptoms include chills, malaise, sore throat, increased confusion, rhinorrhea or nasal congestion, myalgia, dizziness, headache, nausea, and diarrhea.

** Residents might have multiple conditions.

One week after testing, the 13 residents who had positive test results and were asymptomatic on the date of testing were reassessed; 10 had developed symptoms and were recategorized as presymptomatic at the time of testing (Table 2). The most common signs and symptoms that developed were fever (eight residents), malaise (six), and cough (five). The mean interval from testing to symptom onset in the presymptomatic residents was 3 days. Three residents with positive test results remained asymptomatic.

**TABLE 2:** Follow-up symptom assessment 1 week after testing for SARS-CoV-2 among 13 residents of a long-term care skilled nursing facility who were asymptomatic on March 13, 2020 (date of testing) and had positive test results — facility A, King County, Washington, March 2020

Symptom status 1 week after testing	No. (%)
Asymptomatic	3 (23.1)
Developed new symptoms	10 (76.7)
Fever	8 (61.5)
Malaise	6 (46.1)
Cough	5 (38.4)
Confusion	4 (30.8)
Rhinorrhea/Congestion	4 (30.8)
Shortness of breath	3 (23.1)
Diarrhea	3 (23.1)
Sore throat	1 (7.7)
Nausea	1 (7.7)
Dizziness	1 (7.7)

Real-time RT-PCR Ct values for both genetic markers among residents with positive test results for SARS-CoV-2 ranged from 18.6 to 29.2 (symptomatic [typical symptoms]), 24.3 to 26.3 (symptomatic [atypical symptoms only]), 15.3 to 37.9 (presymptomatic), and 21.9 to 31.0 (asymptomatic) (Figure). There were no significant differences between the mean Ct values in the four symptom status groups (p = 0.3).

**FIGURE:**
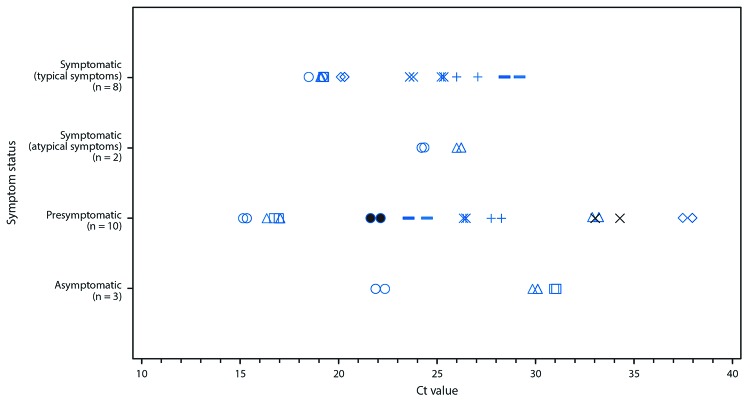
Cycle threshold (Ct) values* for residents of a long-term care skilled nursing facility with positive test results for SARS-CoV-2 by real-time reverse transcription–polymerase chain reaction on March 13, 2020 (n = 23), by symptom status^†,§^ at time of test — facility A, King County, Washington * Ct values are the number of cycles needed for detection of each genetic marker identified by real-time reverse transcription–polymerase chain reaction testing. A lower Ct value indicates a higher amount of viral RNA. Paired values for each resident are depicted using a different shape. Each resident has two Ct values for the two genetic markers (N1 and N2 nucleocapsid protein gene regions). ^†^ Typical symptoms include fever, cough, and shortness of breath. ^§^ Atypical symptoms include chills, malaise, sore throat, increased confusion, rhinorrhea or nasal congestion, myalgia, dizziness, headache, nausea, and diarrhea.

## Discussion

Sixteen days after introduction of SARS-CoV-2 into facility A, facility-wide testing identified a 30.3% prevalence of infection among residents, indicating very rapid spread, despite early adoption of infection prevention and control measures. Approximately half of all residents with positive test results did not have any symptoms at the time of testing, suggesting that transmission from asymptomatic and presymptomatic residents, who were not recognized as having SARS-CoV-2 infection and therefore not isolated, might have contributed to further spread. Similarly, studies have shown that influenza in the elderly, including those living in SNFs, often manifests as few or atypical symptoms, delaying diagnosis and contributing to transmission (*6*–*8*). These findings have important implications for infection control. Current interventions for preventing SARS-CoV-2 transmission primarily rely on presence of signs and symptoms to identify and isolate residents or patients who might have COVID-19. If asymptomatic or presymptomatic residents play an important role in transmission in this population at high risk, additional prevention measures merit consideration, including using testing to guide cohorting strategies or using transmission-based precautions for all residents of a facility after introduction of SARS-CoV-2. Limitations in availability of tests might necessitate taking the latter approach at this time.

Although these findings do not quantify the relative contributions of asymptomatic or presymptomatic residents to SARS-CoV-2 transmission in facility A, they suggest that these residents have the potential for substantial viral shedding. Low Ct values, which indicate large quantities of viral RNA, were identified for most of these residents, and there was no statistically significant difference in distribution of Ct values among the symptom status groups. Similar Ct values were reported in asymptomatic adults in China who were known to transmit SARS-CoV-2 (*9*). Studies to determine the presence of viable virus from these specimens are currently under way.

SNFs have additional infection prevention and control challenges compared with those of assisted living or independent living long-term care facilities. For example, SNF residents might be in shared rooms rather than individual apartments, and there is often prolonged and close contact between residents and health care providers related to the residents’ medical conditions and cognitive function. The index patient in this outbreak was a health care provider, which might have contributed to rapid spread in the facility. In addition, health care personnel in all types of long-term care facilities might have limited experience with proper use of PPE. Symptom ascertainment and room isolation can be exceptionally challenging in elderly residents with neurologic conditions, including dementia. In addition, symptoms of COVID-19 are common and might have multiple etiologies in this population; 24.5% of facility A residents with negative test results for SARS-CoV-2 reported typical or atypical symptoms.

The findings in this report are subject to at least two limitations. First, accurate symptom ascertainment in persons with cognitive impairment and other disabilities is challenging; however, this limitation is estimated to be representative of symptom data collected in most SNFs, and thus, these findings might be generalizable. Second, because this analysis was conducted among residents of an SNF, it is not known whether findings apply to the general population, including younger persons, those without underlying medical conditions, or similarly aged populations in the general community.

This analysis suggests that symptom screening could initially fail to identify approximately one half of SNF residents with SARS-CoV-2 infection. Unrecognized asymptomatic and presymptomatic infections might contribute to transmission in these settings. During the current COVID-19 pandemic, SNFs and all long-term care facilities should take proactive steps to prevent introduction of SARS-CoV-2, including restricting visitors except in compassionate care situations, restricting nonessential personnel from entering the building, asking staff members to monitor themselves for fever and other symptoms, screening all staff members at the beginning of their shift for fever and other symptoms, and supporting staff member sick leave, including for those with mild symptoms (*3*). Once a facility has a case of COVID-19, broad strategies should be implemented to prevent transmission, including restriction of resident-to-resident interactions, universal use of facemasks for all health care personnel while in the facility, and if possible, use of CDC-recommended PPE for the care of all residents (i.e., gown, gloves, eye protection, N95 respirator, or, if not available, a face mask) (*3*). In settings where PPE supplies are limited, strategies for extended PPE use and limited reuse should be employed (*4*). As testing availability improves, consideration might be given to test-based strategies for identifying residents with SARS-CoV-2 infection for the purpose of cohorting, either in designated units within a facility or in a separate facility designated for residents with COVID-19. During the COVID-19 pandemic, collaborative efforts are crucial to protecting the most vulnerable populations.

**Summary**

**What is already known about this topic?**

Once SARS-CoV-2 is introduced in a long-term care skilled nursing facility (SNF), rapid transmission can occur.

**What is added by this report?**

Following identification of a case of coronavirus disease 2019 (COVID-19) in a health care worker, 76 of 82 residents of an SNF were tested for SARS-CoV-2; 23 (30.3%) had positive test results, approximately half of whom were asymptomatic or presymptomatic on the day of testing.

**What are the implications for public health practice?**

Symptom-based screening of SNF residents might fail to identify all SARS-CoV-2 infections. Asymptomatic and presymptomatic SNF residents might contribute to SARS-CoV-2 transmission. Once a facility has confirmed a COVID-19 case, all residents should be cared for using CDC-recommended personal protective equipment (PPE), with considerations for extended use or reuse of PPE as needed.

## References

[1] CDC COVID-19 Response Team. Severe outcomes among patients with coronavirus disease 2019 (COVID-19)—United States, February 12–March 16, 2020. MMWR Morb Mortal Wkly Rep 2020;69:343–6. 10.15585/mmwr.mm6912e2 32214079 PMC7725513

[2] McMichael TM, Clark S, Pogosjans S, COVID-19 in a long-term care facility—King County, Washington, February 27–March 9, 2020. MMWR Morb Mortal Wkly Rep 2020;69:339–42. 10.15585/mmwr.mm6912e1 32214083 PMC7725515

[3] CDC. Preparing for COVID-19: long-term care facilities, nursing homes. Atlanta, GA: US Department of Health and Human Services, CDC; 2020. https://www.cdc.gov/coronavirus/2019-ncov/healthcare-facilities/prevent-spread-in-long-term-care-facilities.html

[4] CDC. Strategies for optimizing the supply of PPE. Atlanta, GA: US Department of Health and Human Services, CDC; 2020. https://www.cdc.gov/coronavirus/2019-ncov/hcp/ppe-strategy/index.html

[5] CDC. Interim guidelines for collecting, handling, and testing clinical specimens from persons for coronavirus disease 2019 (COVID-19). Atlanta, GA: US Department of Health and Human Services, CDC; 2020. https://www.cdc.gov/coronavirus/2019-nCoV/lab/guidelines-clinical-specimens.html

[6] Lam PP, Coleman BL, Green K, Predictors of influenza among older adults in the emergency department. BMC Infect Dis 2016;16:615. 10.1186/s12879-016-1966-4 27793117 PMC5084347

[7] Lansbury LE, Brown CS, Nguyen-Van-Tam JS. Influenza in long-term care facilities. Influenza Other Respir Viruses 2017;11:356–66. 10.1111/irv.12464 28691237 PMC5596516

[8] Sayers G, Igoe D, Carr M, High morbidity and mortality associated with an outbreak of influenza A(H3N2) in a psycho-geriatric facility. Epidemiol Infect 2013;141:357–65. 10.1017/S0950268812000659 22672856 PMC9167657

[9] Zou L, Ruan F, Huang M, SARS-CoV-2 viral load in upper respiratory specimens of infected patients. N Engl J Med 2020;382:1177–9. 10.1056/NEJMc2001737 32074444 PMC7121626

